# Surgery with curative-intent in patients treated with first-line chemotherapy plus bevacizumab for metastatic colorectal cancer First BEAT and the randomised phase-III NO16966 trial

**DOI:** 10.1038/sj.bjc.6605259

**Published:** 2009-09-29

**Authors:** A Okines, O del Puerto, D Cunningham, I Chau, E Van Cutsem, L Saltz, J Cassidy

**Affiliations:** 1 Department of Medicine, The Royal Marsden Hospital NHS Foundation Trust, Sutton, Surrey, UK; 2 F Hoffman La Roche Ltd, Welwyn Garden City, UK; 3 Digestive Oncology Unit, University Hospital, Gasthuisberg, Leuven, Belgium; 4 Memorial Sloan Kettering Cancer Center, New York City, NY, USA; 5 Cancer Research UK, Glasgow, UK

**Keywords:** metastatic, colorectal cancer, bevacizumab, curative-intent, R0 metastasectomy

## Abstract

**Background:**

Complete resection of metastases can result in cure for selected patients with metastatic colorectal cancer.

**Methods:**

First BEAT evaluated the safety of bevacizumab with first-line chemotherapy in 1914 patients. Prospectively collected data from 225 patients who underwent curative-intent surgery were analysed, including an exploratory comparison of resection rate in patients treated with different regimens. NO16966 compared efficacy of oxaliplatin-based chemotherapy plus bevacizumab or placebo in 1400 patients. A retrospective analysis of resection rate was undertaken.

**Results:**

In First BEAT, 225 out of 1914 patients (11.8%) underwent curative-intent surgery at median 64 days (range 42–100) after the last dose of bevacizumab. R0 resection was achieved in 173 out of 225 patients (76.9%). There were no surgery-related deaths and serious post-operative complications were uncommon, with grade 3/4 bleeding and wound-healing events reported in 0.4% and 1.8%, respectively. Resection rates were highest in patients receiving oxaliplatin-based combination chemotherapy (*P*=0.002), possibly confounded by patient selection. In NO16966, 44 out of 699 patients treated with bevacizumab (6.3%) and 34 out of 701 patients treated with placebo (4.9%) underwent R0 metastasectomy (*P*=0.24).

**Conclusions:**

The rate of serious post-operative complications in First BEAT was comparable to historical controls without bevacizumab. In NO16966, there were no statistically significant differences in resection rates or overall survival in patients treated with bevacizumab *vs* placebo.

The addition of oxaliplatin and irinotecan to 5-fluorouracil (5-FU) in metastatic colorectal cancer (mCRC) has improved patient survival and the chance of downsizing initially unresectable metastatic disease, to allow curative-intent surgery. Although the optimal management of liver metastases which are resectable at presentation has not been definitively established, the prospective, randomised controlled European Organisation for Research and Treatment of Cancer (EORTC), Belgium/Intergroup trial 40983 of peri-operative 5-FU and oxaliplatin (FOLFOX4) *vs* surgery alone demonstrated a statistically significant difference in progression-free survival (PFS) in the subgroups of eligible and resected patients, although not the intention to treat (ITT) population (Nordlinger *et al*, 2008), supporting some role for peri-operative chemotherapy. Unfortunately, the trial did not meet the primary endpoint and neither distinguished between synchronous *vs* metachronous presentations, nor stratified for the length of disease-free interval in the latter group. However, peri-operative chemotherapy is a widely accepted strategy, in particular for synchronous presentations, or metachronous presentations occurring soon after treatment of the primary tumour. An alternative strategy for patients with resectable liver metastases is the use of adjuvant chemotherapy, supported by a recently published combined analysis of the Federation Francophone de Cancerologie Digestive (FFCD) Trial 9002 and the EORTC/National Cancer Institute of Canada Clinical Trials Group (NCICCTG), Canada/Gruppo Italiano di Valutazione Interventi in Oncologia phase-III trials. The analysis included 278 patients and showed a moderate, but non-statistically significant benefit for adjuvant bolus 5-FU plus leucovorin over surgery alone for PFS (27.9 *vs* 18.8 months, hazard ratio (HR) 1.32; 95% confidence interval (CI), 1.00–1.76; *P*=0.058) and overall survival (OS) (62.2 *vs* 47.3 months, HR 1.32; 95% CI, 0.95–1.82; *P*=0.095) (Mitry *et al*, 2008).

A newer treatment paradigm has been proposed for highly selected patients with unresectable liver-only metastases in such patients, primary chemotherapy may allow a proportion, in whom adequate downsizing is achieved, to undergo subsequent resection (Leichman, 2007). These patients, by the estimates of some investigators, may comprise up to a quarter of patients presenting with mCRC (Adam *et al*, 2001, 2004). In one study, patients who underwent hepatic resection after conversion chemotherapy had a reduced OS compared with those with primary resectable disease (Adam *et al*, 2004); however, with a median follow up of 48.7 months, 18% remained free of disease, and so are potentially cured (Watkins *et al*, 2007). The median OS from mCRC treated with 5-FU, oxaliplatin and irinotecan has reached over 20 months, whether given concomitantly (Falcone *et al*, 2007) or sequentially (Tournigand *et al*, 2004), but despite this, <5% of unresectable patients will live up to 5-years with chemotherapy alone (Chua and Cunningham, 2006). In contrast, the reported 5-year survival of the highly selected group of patients with initially unresectable liver-only disease treated with conversion chemotherapy then surgery ranges from 33–50% (Giacchetti *et al*, 1999; Adam *et al*, 2001, 2004). Inevitably, some patients will not benefit from metastasectomy after conversion chemotherapy and identifying these patients is vital for preventing futile surgery.

Prospective evaluation of conversion chemotherapy for patients with liver-only, primarily unresectable disease has been undertaken in the phase-II setting, with response rates (RR) of 48–71% and R0 resection rate of 26–41% in these selected populations (Pozzo *et al*, 2004; De La Cámara *et al*, 2004; Alberts *et al*, 2005; Ychou *et al*, 2007), however, high rates of recurrence of up to 73% were reported (Alberts *et al*, 2005; Giacchetti *et al*, 1999). Resection rate has also been prospectively evaluated within phase-II and -III trials in patients with any-site mCRC, with RR of 33–66% and R0 resection rates of up to 15% reported, despite the unselected population (Tournigand *et al*, 2004, 2006; Delaunoit *et al*, 2005; Falcone *et al*, 2007).

The addition of bevacizumab, to first and second line chemotherapy for mCRC has demonstrated statistically significant improvements in PFS (Kabbinavar *et al*, 2003, 2005; Hurwitz *et al*, 2004; Giantonio *et al*, 2007; Saltz *et al*, 2008), and statistically significant improvement in OS in some studies (Hurwitz *et al*, 2004; Giantonio *et al*, 2007). However, there are currently no data evaluating the role of bevacizumab added to chemotherapy in the peri-operative setting. We present here our evaluation of this topic in two large prospective trials.

## Materials and methods

Two large international multicentre studies were included in this analysis. First bevacizumab expanded access trial (BEAT) added bevacizumab to investigator's choice fluoropyrimidine-based chemotherapy in the first-line treatment of mCRC. This international trial recruited 1965 patients from 41 countries between June 2004 and February 2006. Eligible patients were aged at least 18-years old, had histologically confirmed mCRC, had not received chemotherapy for mCRC previously, were of ECOG performance status 0–1, with adequate bone marrow, liver and renal function, without significant proteinuria and were scheduled to receive first-line fluoropyrimidine-based chemotherapy. Patients who had undergone major surgery within 28 days, had a serious non-healing wound or ulcer, a bleeding diathesis, coagulopathy or were on therapeutic anticoagulation, had uncontrolled hypertension or clinically significant cardiovascular disease were excluded from the study.

Safety was the primary endpoint of the trial. This included the incidence of all reported adverse or serious adverse events, serious adverse events related to bevacizumab and the targeted adverse events of gastrointestinal perforation, wound healing complications, hypertension, proteinuria, bleeding and thromboembolism. Time to disease progression; defined as the time period from the start of first-line therapy to investigator assessed disease progression, and OS; defined as the time period from the start of first-line therapy to death, were secondary endpoints.

Data for secondary resections (metastasectomy) were prospectively collected in the overall (ITT) population, defined as all patients who received at least one dose of the study drug, bevacizumab. The safety of bevacizumab in all patients undergoing secondary resection was also prospectively examined.

The NO16966 study was designed to show non-inferiority of capecitabine plus oxaliplatin (XELOX) to FOLFOX4 after the presentation of the pivotal trial by Hurwitz *et al*, showing a survival benefit from adding bevacizumab to first-line combination chemotherapy with irinotecan and bolus 5-FU/leucovorin (IFL) for mCRC, the trial was amended to a two-by-two design to assess the addition of bevacizumab to each combination. Between February 2004 and February 2005, 1401 patients were randomised to the four arms of the trial, which met the primary endpoints of non-inferiority of XELOX to FOLFOX for PFS and superiority of chemotherapy plus bevacizumab to chemotherapy plus placebo for PFS. The ITT population (*n*=1400) was defined as all patients who signed the trial consent form and were randomised. Eligibility criteria for this trial have been published previously.

Secondary resection rate was not a predefined endpoint of the NO16966 trial and as such, the trial was not powered to detect a difference in patients treated with or without bevacizumab, but these data have been retrospectively collected and analysed for the ITT population. Bevacizumab-related adverse events were prospectively collected in the study, but the rate of peri-operative complications was not.

For both studies, the protocol was reviewed by an independent review body and all patients were required to give written, informed consent.

### Statistical methods

#### First BEAT

Resection rates were calculated for the ITT population, for curative-intent surgery (the site of surgery was the only site of disease), R0 resection (complete resection with no residual disease), all hepatic surgery performed, curative-intent hepatic surgery and R0 resection rate.

Resection rates were also calculated for the exploratory subgroups of patients treated with oxaliplatin-based chemotherapy regimens and irinotecan-based regimens. Fisher's exact test was used to compare resection rates. Kaplan–Meier survival curves were generated for OS in the subgroups of patients undergoing curative-intent hepatic resection, those undergoing R0 hepatic resection and the remainder of the ITT population. In addition, survival curves were generated for the exploratory subgroup of patients with liver-only metastatic disease who underwent R0 resection *vs* those who did not.

The log-rank test was used to compare PFS and OS in those undergoing any surgery, all curative-intent surgery, R0 resections, hepatic metastasectomy, curative-intent hepatic metastasectomy and R0 hepatic metastasectomy compared with those that did not, in the ITT population and the subgroup of patients with liver-only disease.

#### NO16966

*χ*^2^ test comparisons were used for the rates of curative (R0 resection) surgery and curative liver surgery. The log-rank test was used to compare PFS in patients randomised to receive bevacizumab, *vs* those receiving placebo. No additional statistical testing was applied to the adverse event rates for the bevacizumab comparison, which have been previously published. Complication rates in patients who underwent surgery were not collected during the study.

## Results

### First BEAT

Baseline characteristics for the 1914 patients evaluable for the final analysis in February 2008 are summarised in Table 1 .

### ITT population resection rate

Table 2 shows that 225 out of 1914 patients (11.8%) underwent surgery with curative-intent, of whom R0 resection was achieved in 173 out of 225 patients (76.9%). The median duration of treatment before curative-intent surgery was 148 days (range 85–227 days). Surgery was undertaken at a median of 64 days after the last dose of bevacizumab (range 42–100 days).

The surgery comprised of curative-intent hepatic metastasectomy in 145 cases (7.6%), with R0 resection reported in 114 out of 145 patients (78.6%). The type of curative-intent surgery undertaken in the remaining 80 out of 225 patients were not collected.

Of patients who received oxaliplatin-based combination chemotherapy (with 5-FU or capecitabine) 153 out of 949 (16.1%) underwent surgery with curative-intent, whereas 64 out of 662 (9.7%) of patients treated with irinotecan based combinations underwent surgery. In an exploratory comparison of these numbers, the difference is statistically significant (*P*=0.002). R0 resection was achieved in 116 out of 153 patients (75.8%) that received oxaliplatin-based chemotherapy and 49 out of 64 patients (76.6%) that received irinotecan-based combinations.

### ITT population OS

**Figure 1:**
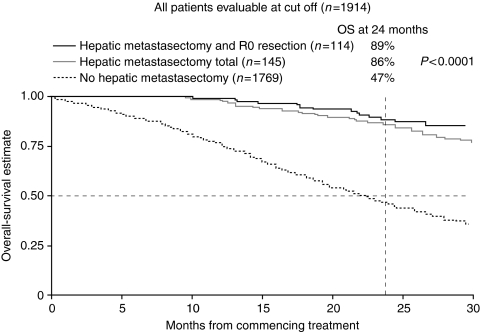
Kaplan–Meier estimate for overall survival in patients undergoing curative-intent hepatic resection, curative hepatic resection with no residual disease (R0) and the remainder of the intention to treat (ITT) population in First bevacizumab expanded access trial (BEAT).

The median OS is 21.4 months (95% CI 20.5–22.4 months) in the 1769 patients who did not undergo curative-intent surgery and has not been reached in the 145 patients who underwent curative-intent hepatic surgery (HR 0.24, 95% CI 0.17–0.34, *P*<0.001) or the 114 patients in whom R0 resection was achieved (HR 0.15, 95% CI 0.10–0.25, *P*<0.001). The Kaplan–Meier curves for patients undergoing curative-intent hepatic surgery, those who achieved R0 resection and those who did not undergo curative-intent surgery are shown in Figure 1.

### Patients with metastatic disease confined to the liver; resection rate

In the sub-population of patients with liver-only metastatic disease (*n*=704), 107 patients (15.2%) underwent curative-intent hepatic metastasectomy, which was a R0 resection in 85 out of 107 patients (79.4%). It should be noted, however, that neither FIRST BEAT nor NO16966 required formal multidisciplinary evaluation for resectability before entrance; it is likely that a portion of these patients would have been deemed resectable from the outset at some specialised centres.

Table 3 shows the exploratory comparison of resection rate in the subgroups of patients with liver-only metastases treated with oxaliplatin or irinotecan based chemotherapy. Curative-intent hepatic resection rate was higher in 350 patients treated with oxaliplatin-based combination chemotherapy (71 out of 350 patients; 20.3%) than in those treated with irinotecan combination chemotherapy (33 out of 230patients; 14.5%) although the difference did not reach statistical significance (*P*=0.077).

### Patients with metastatic disease confined to the liver OS

**Figure 2:**
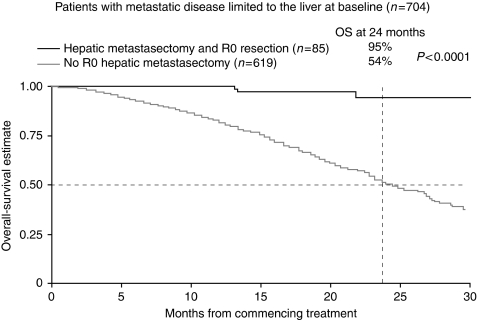
Kaplan–Meier survival curves for all patients with liver-only disease undergoing R0 hepatic resections *vs* those with liver only disease that did not in First bevacizumab expanded access trial (BEAT).

OS was significantly longer in patients who underwent hepatic resections compared with those who did not (log rank *P*<0.001). The OS for all patients with liver-only disease within First-BEAT was 25.2 months and has not yet been reached in those undergoing curative-intent metastasectomy. The 2-year OS rate in all patients with liver-only disease is 54%, compared with 89% in patients undergoing curative-intent resection and 94% in those who achieved R0 resection. The Kaplan–Meier survival curves for patients with liver-only disease undergoing R0 resection *vs* those who did not are shown in Figure 2.

### NO16966

Baseline characteristics for the ITT population are summarised in Table 1.

### Resection rate

In the ITT population (*n*=1400), 44 out of 699 (6.3%) patients receiving bevacizumab plus chemotherapy underwent curative (R0) resection, compared with 34 out of 701 (4.9%) patients receiving chemotherapy plus placebo, however, this difference was not statistically significant (*P*=0.24). Similarly, when analyzing the subgroup of patients with liver-only disease at baseline, 26 out of 211 (12.3%) patients of those receiving bevacizumab underwent curative surgery compared with 24 out of 207 (11.6%) patients receiving placebo (*P*=0.81). These data are summarised in Tables 2 and 3.

### Overall survival

Of the 78 patients undergoing R0 resection within the trial, 2-year OS was not statistically different in patients receiving bevacizumab (90.9%), compared with those receiving placebo (82.3%). Two-year OS in patients not undergoing R0 resection was 39.6% in patients receiving bevacizumab and 37.9% in those randomised to chemotherapy plus placebo.

### Safety

Table 4 shows bevacizumab related grade 3/4 adverse events for patients treated within NO16966 and First BEAT, and specific surgical complications that were prospectively collected from patients undergoing curative-intent resections in First BEAT. It should be noted that there were no surgery-related deaths (deaths due to events that developed within 30 days of surgery).

## Discussion

First BEAT prospectively collected safety data from patients undergoing surgery and is the largest data set using a targeted agent in this setting. Data from the randomised phase-III NO16966 trial were collected retrospectively, therefore, must be interpreted with more caution.

First BEAT reported a 6% R0 hepatic resection rate in the unselected population and 12.1%, when selecting patients with liver-only disease. In the NO16966 study, there was no significant difference in R0 resection rate in patients treated with bevacizumab compared with placebo (6.3 *vs* 4.9%, *P*=0.24). A major limitation of the analysis of resection rate in both studies is that resectability was not prospectively defined. It is conceivable that some patients may have had resectable disease from the outset, especially if reviewed by an experienced hepatobiliary surgeon at a tertiary referral centre.

The optimal regimen for patients with potentially resectable disease is yet to be defined, but a strong correlation between response to chemotherapy and subsequent resection rate has been described (Folprecht *et al*, 2005). An exploratory subgroup analysis of the First BEAT data demonstrated that a higher proportion of patients treated with bevacizumab plus oxaliplatin-based chemotherapy than bevacizumab plus an irinotecan-based regimen underwent curative-intent surgery (16.1% *vs* 9.7%). However, the resection rate within the oxaliplatin-treated subgroup in First BEAT is substantially higher than seen in N016966, which could suggest that First BEAT investigators chose to use oxaliplatin-based regimens in patients with potentially resectable disease. There are also differences in the baseline characteristics of the patient populations of each study, which may have contributed to the difference in resection rates. For example, more patients in First BEAT had a single site of metastatic disease (61% compared with 41 and 42% in the bevacizumab and placebo arms, respectively).

In First BEAT, overall survival was longer in patients who underwent hepatic metastasectomy compared with those who did not. A limitation of this comparison is that at the start of treatment, all patients received chemotherapy, and therefore there were no patients in the surgery group. Hence, the estimates and comparisons are biased, as patients who eventually underwent surgery have an initial guaranteed survival time.

Although bevacizumab improved the response rate when added to IFL (Hurwitz *et al*, 2004), which has since been superseded by the FOLFIRI regimen, it did not improve the response at all when added to XELOX or FOLFOX (Saltz *et al*, 2008). Thus, although the choice of chemotherapy regimen may be key to maximising resection rate with bevacizumab combinations, the choice to be made is unclear. A superior response rate has been reported with the FOLFOXIRI regimen (66% *vs* 41% with FOLFIRI) in the first-line setting, with a corresponding increase in R0 resection rate, reported as 36% in a subgroup of patients with liver-only metastatic disease (Falcone *et al*, 2007). However, a second phase-III trial of the regimen reported a more modest response rate of 43% from the combination (33.6% with FOLFIRI), possibly due to the lower doses of all three agents used.

An apparent increase in steatohepatitis and a subsequently increased 90-day mortality after liver resection has been reported with irinotecan given before liver surgery (Vauthey *et al*, 2006). Oxaliplatin is also known to affect the liver, causing sinusoidal dilatation in 19% of cases in the same series (Vauthey *et al*, 2006). It is interesting to note that in a retrospective series of 105 patients treated with oxaliplatin-based chemotherapy with or without bevacizumab, the investigators reported a lower incidence and severity of sinusoidal dilatation in patients receiving bevacizumab (*P*<0.01) (Ribero *et al*, 2007). The authors suggested that this may be because of VEGF blockade-mediated down-regulation of matrix-metalloproteinase-9, which is known to be involved in sinusoidal dilatation. A recently published non-randomised phase-II study of bevacizumab with capecitabine and oxaliplatin in 56 patients with potentially resectable liver metastases reported one case of abnormal liver function/regeneration due to steatohepatitis, (Gruenberger *et al*, 2008) which is consistent with the observation that major perturbations in liver function or liver regeneration with chemotherapy of moderate duration are rare.

The optimal timing of surgery after chemotherapy alone is again unresolved. In the EORTC Intergroup Trial 40983, surgery was planned for 2–5 weeks after the last administration of chemotherapy and took place a median of 4.1 weeks after chemotherapy (range 2–16.4 weeks) (Nordlinger *et al*, 2008). Reversible peri-operative complications were more frequent in the chemotherapy arm (25% *vs* 16%, *P*=0.04) with notable increases in biliary fistulae, hepatic failure and intra-abdominal infections, but no difference in bleeding, venous thromboembolism or post-operative death rates. Whether the increase in complications related to the timing of surgery relative to chemotherapy, or simply the exposure to chemotherapy itself, cannot be determined from these data, although data from a retrospective series of 750 patients undergoing hepatic resection, suggest that the rate of complications is highest in patients undergoing surgery within 4 weeks of chemotherapy (Welsh *et al*, 2007).

More caution is generally applied when bevacizumab is used before or after surgery because of potential effects on bleeding, wound-healing and liver regeneration. In an analysis of pooled data from two randomised studies, the reported wound-healing complication rate after an unplanned major surgery during treatment was 13% for patients treated with 5-FU and bevacizumab compared with 3.4% in patients treated with chemotherapy alone (Scappaticci *et al*, 2005). However, in patients that had undergone major surgery 28–60 days before commencing bevacizumab, a significant difference in wound-healing complications was not observed in the bevacizumab and control arms (1.3% *vs* 0.5%) (Scappaticci *et al*, 2005), suggesting that the risk of this complication can be reduced by careful management of the timing of surgery in relation to bevacizumab. In First BEAT, surgery took place at a median of 64 days after the last dose of bevacizumab, which could be considered as a conservative approach, but was associated with a relatively low rate of serious complications. Results from a small, non-randomised study suggests that a shorter time period between bevacizumab and surgery may be reasonable; this phase-II trial using a 5-week delay reported a 21% total complications rate after liver surgery (less than reported in the EORTC 40983 trial (Nordlinger *et al*, 2008)), with blood transfusions required in 6% of the patients and no peri-operative mortality (Gruenberger *et al*, 2008).

There are fewer safety concerns with the addition of the EGFR monoclonal antibody, cetuximab, to neo-adjuvant chemotherapy. Data from a pre-planned analysis in the phase-III CRYSTAL trial demonstrated potential efficacy of adding cetuximab to downsize initially unresectable disease, with R0 resections achieved in 4.3% of patients treated with FOLFIRI plus cetuximab, compared with 1.5% in those treated with FOLFIRI alone (*P*=0.0034) and 9.8% *vs* 4.5% R0 resections respectively, in an exploratory subgroup analysis of patients with liver-only disease (Van Cutsem *et al*, 2009). These resection rates are lower, although comparable, to those reported here, however, the resection rates in the chemotherapy arm alone are notably lower than in the placebo arm of NO16966 (4.9% of the ITT population, 11.6% of patients with liver-only disease), possibly due to the different cytotoxic chemotherapy regimens. It should be noted that cetuximab has recently been recommended by the UK National Institute for Clinical Excellence (NICE) for use in combination with FOLFOX or FOLFIRI in patients with K-ras wild-type and unresectable, liver-only mCRC.

## Conclusions

The rate of serious post-operative complications in First BEAT was acceptable, with resection rates consistent with those reported in the recent literature. The contribution of bevacizumab is difficult to evaluate outside a randomised controlled trial. In NO16966, there was no statistically significant difference in resection rate or the OS in patients treated with bevacizumab *vs* placebo, however, the numbers of patients undergoing resection in this trial was small; thus the ability of this trial to detect a difference in resection rates would be quite limited.

## Figures and Tables

**Table 1:** Baseline characteristics of patients enrolled in the First BEAT and NO16966 trials

	**First BEAT trial (n=1914)**	**NO16966**	
		**FOLFOX or XELOX + placebo (n=701)**	**FOLFOX or XELOX + bevacizumab (n=699)**
*Sex* (%)			
Male	58	56	60
Female	42	44	40
			
*Age*			
Median	59	60 (FOLFOX/placebo)	60 (FOLFOX/bev)
		61 (XELOX/placebo)	61 (XELOX/bev)
			
*ECOG PS* (%)			
0	65	60	58
1	34	40	41
			
*Primary tumour site* (%)			
Colon	62	66	66
Rectum	27	26	26
Colon and rectum	11	8	9
			
*Previous adjuvant therapy* (%)			
Yes	38	25	23
No	62	75	77
			
*Number of metastatic sites* (%)			
1	61	42	41
>1	39	57	59

Abbreviations: BEAT=bevacizumab expanded access trial; ECOG=Eastern Cooperative Oncology Group; FOLFOX4=5-FU and oxaliplatin; XELOX=capecitabine plus oxaliplatin.

**Table 2:** Patients undergoing resections within the First BEAT and N016966 trials

	**No. of patients undergoing surgery with curative intent (%)**	**No. of patients undergoing R0 resection (%)**	**% Of curative- intent resections that were R0**	**Hepatic resections with curative intent (%)**	**R0 hepatic resections (%)**	**% Of hepatic resections that were R0**
*First BEAT*						
All evaluable patients (ITT population) *n*=1914	225 (11.8%)	173 (9.0)	76.9	145 (7.6)	114 (6.0)	78.6
Patients receiving oxaliplatin-based chemotherapy *n*=949	153 (16.1)	116 (12.2)	75.8	99 (10.4)	76 (8.0)	76.8
Patients receiving irinotecan-based chemotherapy *n*=662	64 (9.7)	49 (7.4)	76.6	43 (6.5)	34 (5.1)	79.1
						
*NO16966*						
XELOX or FOLFOX4 + bevacizumab *n*=699	59 (8.4)	44 (6.3)	74.6	—	—	—
XELOX or FOLFOX4 + placebo *n*=701	43 (6.1)	34 (4.9)	79.1	—	—	—

Abbreviations: BEAT=bevacizumab expanded access trial; FOLFOX4=5-FU and oxaliplatin; ITT=intention to treat; XELOX=capecitabine plus oxaliplatin.

**Table 3:** Patients with liver-only disease within the First BEAT and NO16966 trials

	**All patients undergoing hepatic resection (%)**	**Curative-intent hepatic resections (%)**	**R0 hepatic resections (%)**
*First BEAT*			
All patients with liver-only disease *n*=704	132 (18.8%)	107 (15.2%)	85 (12.1%)
Patients receiving oxaliplatin-based chemotherapy *n*=350	85 (24.3%)	71 (20.3%)	54 (15.4%)
Patients receiving irinotecan-based chemotherapy *n*=230	43 (18.7%)	33 (14.3%)	27 (11.7%)
			
*NO16966*			
XELOX or FOLFOX4 + bevacizumab *n*=211			26 (12.3)
XELOX or FOLFOX4 + placebo *n*=207			24 (11.6)

Abbreviations: BEAT=bevacizumab expanded access trial; FOLFOX4=5-FU and oxaliplatin; XELOX=capecitabine plus oxaliplatin.

**Table 4:** Grade 3/4 bevacizumab-related serious adverse events in NO16966 and First BEAT and surgical complications in resected patients in First BEAT

	**Bleeding**	**Wound healing complications**	**GI perforation**	**Hypertension**	**Arterial thrombo-embolism**
*NO16966*					
FOLFOX4 or XELOX + placebo (*n*=675)	8 (1.2%)	2 (0.3%)	2 (0.3%)	8 (1.2%)	7 (1.0%)
FOLFOX4 or XELOX + bevacizumab (*n*=694)	13 (1.9%)	1 (0.1%)	4 (0.6%)	26 (3.7%)	12 (1.7%)
First BEAT (ITT population *n*=1914)	61 (3.2%)	20 (1.0%)	34 (1.8%)	97 (5.1%)	24 (1.3%)
					
*First BEAT*					
Surgical complications in patients undergoing curative-intent metastasectomy (*n*=225)	1 (0.4%) Any grade: 3 (1.3%)	4 (1.8%) Any grade: 11 (4.9%)	5 (2.2%)	16 (7.1%)	3 (1.3%)

Abbreviations: BEAT=bevacizumab expanded access trial; FOLFOX4=5-FU and oxaliplatin; ITT=intention to treat; XELOX=capecitabine plus oxaliplatin.
